# Supplementary material for: Extracellular matrix mediates circulating tumor cell clustering in triple-negative breast cancer metastasis
Source: Nat Commun. 2026 Feb 6;17:1352. doi: 10.1038/s41467-026-69007-w (PMC12881594; doi:10.1038/s41467-026-69007-w)
Supplement: Supplementary file 1 — Supplementary Information [file 41467_2026_69007_MOESM1_ESM.pdf]

Supplementary Information

Extracellular matrix mediates circulating tumor cell clustering in triple-negative breast cancer metastasis

Georg OM Bobkov, Khushali J Patel, Bree M Lege, Rong Zheng, Gad Shaulsky, Matthew J Ellis, Chonghui Cheng

Bobkov et al., Supplementary Figure 1

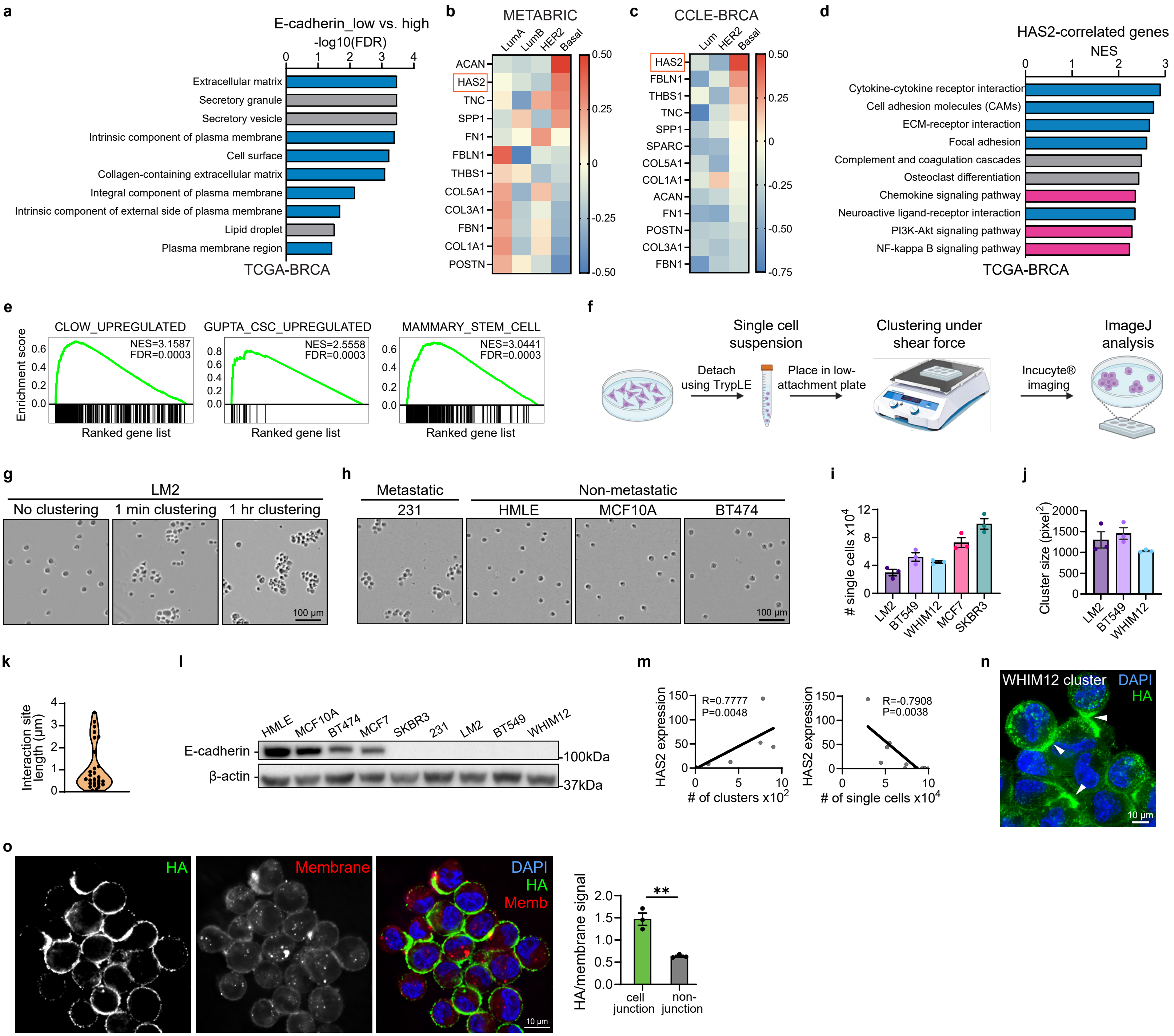

Supplementary Figure 1. HAS2 expression correlates with clustering of TNBC cells.

a, Gene Ontology analysis showing top cellular component terms positively enriched in tumors with low E-cadherin expression (bottom 10%) vs. high E-cadherin expression (top 10%) in the TCGA-BRCA dataset. ECM and plasma membrane terms are shown in blue; other terms are shown in gray. **b,c**, Heatmaps showing expression of ECM-related genes implicated in breast cancer progression by breast cancer subtype in the METABRIC (**b**) and CCLE-BRCA (**c**) datasets. **d**, GSEA showing top KEGG pathways positively enriched in HAS2-correlated genes in the TCGA-BRCA dataset. Ligand-receptor interaction pathways (blue) and cancer-associated signaling pathways (pink) are highlighted. Other pathways are shown in gray. **e**, GSEA plots showing significant positive enrichment of claudin-low, cancer stem cell, and mammary stem cell signatures in HAS2-correlated gene list from the TCGA-BRCA dataset. **f**, Schematic depiction of the *in vitro* cluster assay. Created in BioRender. Bobkov, G. (2026) https://BioRender.com/srzuwrk. **g**, Representative images of LM2 cells after no clustering and 1 min or 1 hr of clustering. See also Fig. 1e for quantification. **h**, Representative images of cells after 1 hr of clustering. Metastatic and non-metastatic breast cell lines are shown. **i**, Quantification of the remaining single cells of each cell line shown in Fig. 1f (n=3 biological replicates). **j**, Quantification of the cluster size of the three clustering cell lines shown in Fig. 1f (n=3 biological replicates). **k**, Quantification of length of interaction site exemplified in Fig. 1h (n=27 cell-cell interaction sites from 3 biological replicates). **l**, Western blot showing E-cadherin protein levels in breast cell lines. β-actin served as a loading control. Source Data are provided as a Source Data file. **m**, Correlation of HAS2 expression with number of clusters (left) and remaining single cells (right). Average values of 3 biological replicates were used to plot each point. *P*-value was determined using Pearson correlation. **n**, Maximum intensity projection of a fixed WHIM12 tumor cell cluster stained for HA (green). White arrowheads indicate examples of HA enrichment. **o**, Left: maximum intensity projection of a fixed LM2 cluster stained for HA (green) and CellBrite® Fix 555 Membrane Stain (red). “Memb” indicates membrane. Right: Quantification of HA signal divided by the corresponding membrane signal for the clusters exemplified on the left (n=3 biological replicates; *P*=0.0037). DAPI (blue) served as a nuclear counterstain. Data are represented as mean ± SEM. Statistical significance: ***P* = <0.01 (unpaired two-sided t test).

Bobkov et al., Supplementary Figure 2

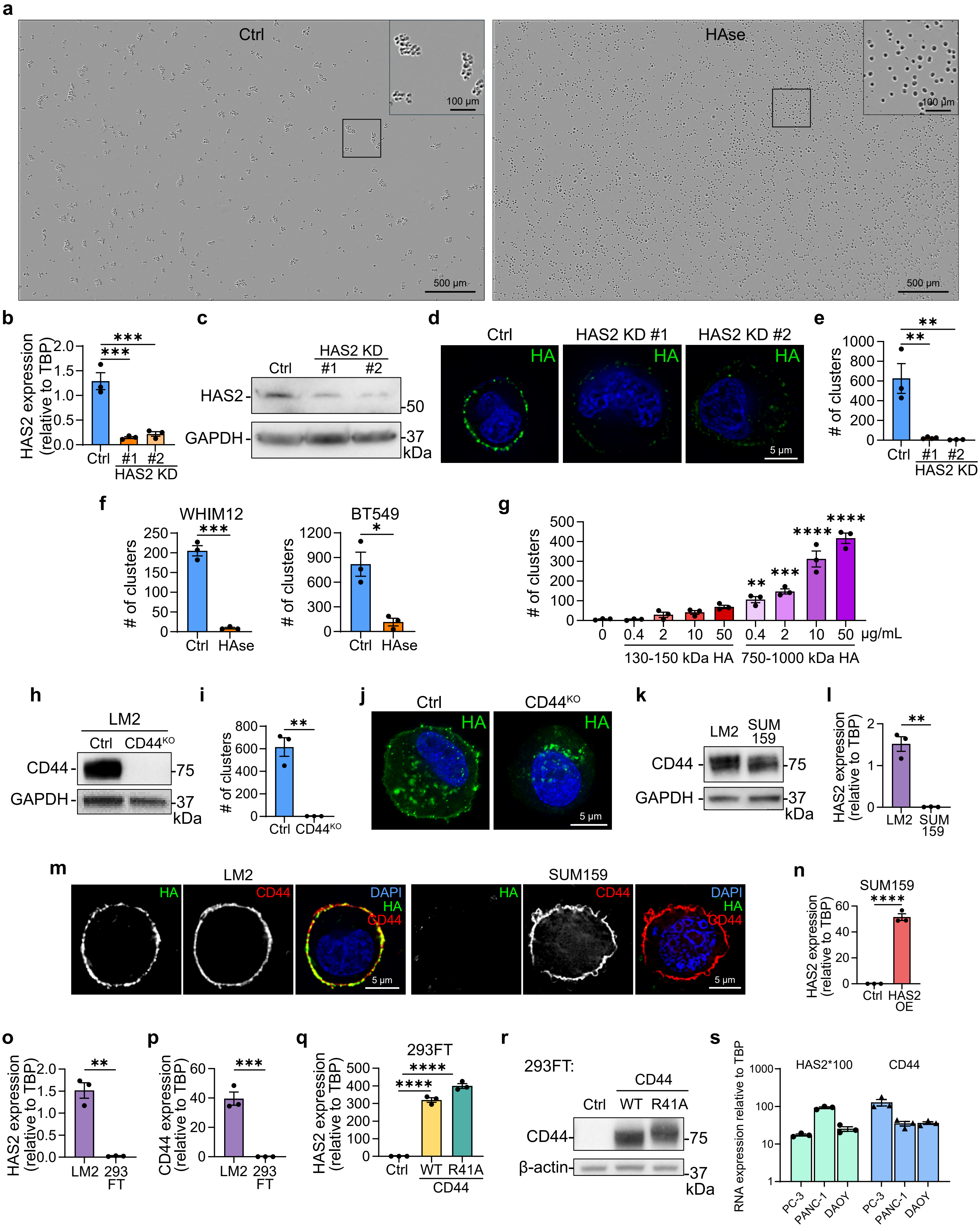

Supplementary Figure 2. HA-CD44 interaction is required and sufficient for clustering.

a, Representative images showing a larger field of view for the clustering images of LM2 control and HAse-treated cells shown in Fig. 2b. Box indicates position of the 2.5-fold magnified region inset on the top right. **b-e**, Data for LM2 control and HAS2 KD cells are shown (n=3 biological replicates). qRT-PCR (**b**; *P*=0.0004, 0.0006) and Western blot (**c**) results showing HAS2 expression. Maximum intensity projection of cells fixed in suspension and stained for HA (green; **d**). Quantification of numbers of clusters formed after 1 hr of clustering (**e**; n=4 biological replicates for KD#1; *P*=0.0014, 0.0018). **f**, Quantification of the number of clusters formed by control and HAse-treated WHIM12 PDX and BT549 cells after 1 hr of clustering (n=3 biological replicates; *P*=0.0001, 0.0101). **g,** Quantification of numbers of clusters formed by LM2 HAS2 KD cells after treatment with increasing concentrations of low-molecular weight (130-150 kDa) and high-molecular weight (750-1000 kDa) HA (n=3 biological replicates; *P*= 0.0083, 0.0003). Cells were pre-incubated with the exogenous HA for 1 hr prior to clustering for 1 hr. **h**, Western blot showing CD44 protein levels of LM2 control and CD44 KO cell lines. **i**, Representative images and quantification of numbers of clusters formed by LM2 control and CD44 KO cells after 1 hr of clustering (n=3 biological replicates; *P*=0.0017). **j**, Maximum intensity projection of LM2 Ctrl and CD44 KO cells fixed in suspension, permeabilized and stained for HA (green). **k**, Western blot showing CD44 protein levels in LM2 and SUM159 cells. **l**, qRT-PCR results showing HAS2 mRNA expression in LM2 and SUM159 cells (n=3 biological replicates; *P*=0.0010). **m**, Maximum intensity projection of LM2 and SUM159 cells stained for HA (green) and CD44 (red). See also Fig. 2e. **n**, qRT-PCR results showing HAS2 mRNA expression in SUM159 Ctrl and HAS2 OE cells (n=3 biological replicates). **o,p**, qRT-PCR results showing HAS2 (**o**; *P*=0.0011) and CD44 (**p**; *P*=0.0009) mRNA expression in LM2 and 293FT cell lines (n=3 biological replicates). **q**, qRT-PCR results showing HAS2 mRNA expression in 293FT control and 293FT co-transfected with either HAS2 + $CD44^{WT}$ or HAS2 + $CD44^{R41A}$ (n=3 biological replicates). **r**, Western blot showing CD44 protein levels in 293FT control and 293FT co-transfected with either HAS2 + $CD44^{WT}$ or HAS2 + $CD44^{R41A}$. **s**, qRT-PCR analysis showing HAS2*100 and CD44 mRNA expression of non-breast cancer cell lines (n=3 biological replicates). All qRT-PCR data is normalized to TBP. β-actin or GAPDH served as loading controls for Western blots. Data are represented as mean ± SEM. Statistical significance: **P* = <0.05; ***P* = <0.01; ****P* = <0.001; *****P* = <0.0001 (unpaired two-sided t test (**b,e,f,i,l,n-p**) or ordinary one-way ANOVA (**g,q**)). DAPI (blue) served as a nuclear counterstain. Source Data (**c, h, k, r**) are provided as a Source Data file.

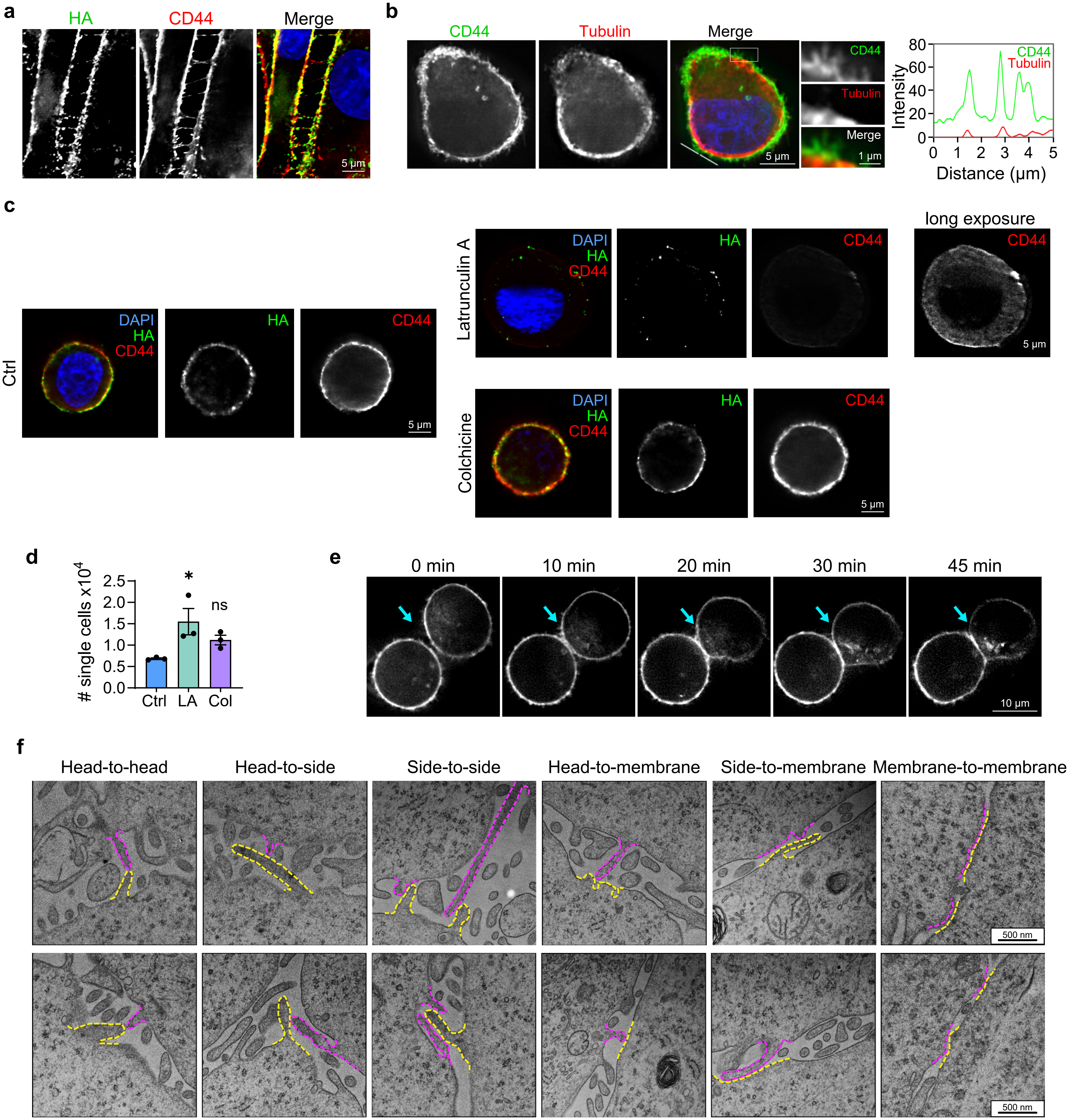
a
HA
CD44
Merge
5 µm
b
CD44
Tubulin
Merge
5 µm
CD44
Tubulin
Merge
1 µm
Intensity
Distance (µm)
c
Ctrl
DAPI
HA
CD44
5 µm
Latrunculin A
long exposure
Colchicine
d
single cells x10^4
2.5
2.0
1.5
1.0
0.5
0.0
Ctrl
LA
Col
*
ns
e
0 min
10 min
20 min
30 min
45 min
10 µm
f
Head-to-head
Head-to-side
Side-to-side
Head-to-membrane
Side-to-membrane
Membrane-to-membrane
500 nm

Supplementary Figure 3. HA-mediated clustering is initiated by actin-based protrusions.

a, Maximum intensity projection of LM2 cells grown on glass coverslips, fixed and stained for HA (green) and CD44 (red). **b**, Left: single optical section of a detached LM2 cell stained for CD44 (green) and tubulin (red). Box indicates the position of the 3-fold magnified region on the right showing protrusions. Line indicates position of the line profile (right) for CD44 (green) and tubulin (red) signals in protrusion. See also Fig. 3d for actin staining of the same cell. **c**, Single optical section of Ctrl, Latrunculin A treated, and Colchicine treated cells stained for HA (green) and CD44 (red). CD44 single channel shows presence/absence of protrusions. High CD44 exposure of Latrunculin A shows smooth, protrusion-free cell body. **d**, Quantification of remaining single cells in LM2 cells treated with Latrunculin A (LA) or Colchicine (Col) after 1 hr of clustering (n=3 biological replicates; *P*=0.0310, 0.2521). See also Fig. 3e. **e**, Live imaging of mid- to late-interaction events between LM2 cells in suspension. Single optical sections are shown; cellular protrusions were visualized using anti-CD44 staining. Minutes that passed since the start of the experiment are displayed above each picture. See also Fig. 3f and Supplementary Movies 1-3. **f**, Transmission electron microscopy pictures depicting the five types of interactions observed in LM2 WT cells after clustering for 5-20 minutes. Images were taken at 5000-15000x magnification. Purple and yellow dotted lines indicate membrane regions involved in each interaction. See also Fig. 3i. Data are represented as mean ± SEM. Statistical significance: ns = not significant; **P* = <0.05 (one-way ANOVA). DAPI (blue) served as a nuclear counterstain.

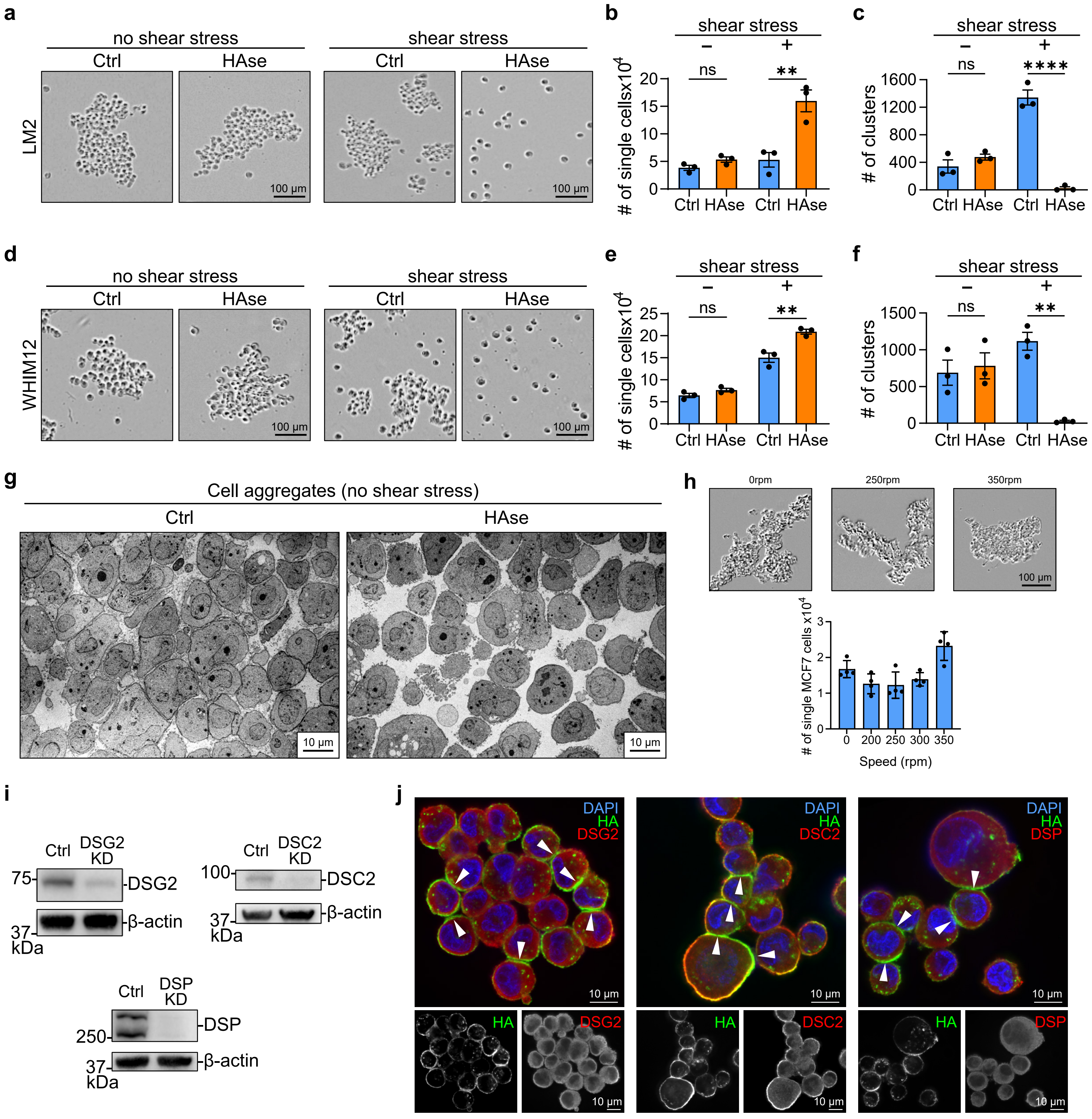
a
no shear stress
shear stress
Ctrl
HAse
Ctrl
HAse
LM2
100 µm
100 µm
b
shear stress
–
+
ns
**
# of single cellsx10^4
20
15
10
5
0
Ctrl HAse Ctrl HAse
c
shear stress
–
+
ns
****
# of clusters
1600
1200
800
400
0
Ctrl HAse Ctrl HAse
d
no shear stress
shear stress
Ctrl
HAse
Ctrl
HAse
WHIM12
100 µm
100 µm
e
shear stress
–
+
ns
**
# of single cellsx10^4
25
20
15
10
5
0
Ctrl HAse Ctrl HAse
f
shear stress
–
+
ns
**
# of clusters
1500
1000
500
0
Ctrl HAse Ctrl HAse
g
Cell aggregates (no shear stress)
Ctrl
HAse
10 µm
10 µm
h
0rpm
250rpm
350rpm
100 µm
# of single MCF7 cells x10^4
3
2
1
0
0 200 250 300 350
Speed (rpm)
i
Ctrl
DSG2 KD
75
DSG2
37
β-actin
kDa
Ctrl
DSC2 KD
100
DSC2
37
β-actin
kDa
Ctrl
DSP KD
250
DSP
37
β-actin
kDa
j
DAPI
HA
DSG2
DAPI
HA
DSC2
DAPI
HA
DSP
10 µm
HA
DSG2
HA
DSC2
HA
DSP

Supplementary Figure 4. HA and desmosomes promote cluster stability and cell survival under shear stress.

a, Representative images of Ctrl and HAse treated LM2 cells after settling for 6 hrs in low-attachment plate (left, no shear stress) and after performing the clustering assay on the 6 hrs-settled aggregates for 1 hr (right, shear stress). **b,c**, Quantification of the number of single cells (**b**; *P*=0.9610, 0.0017) and number of clusters (**c**; *P*=0.8107) from samples shown in **a** (n=3 biological replicates). **d**, Representative images of Ctrl and HAse treated WHIM12 cells after settling for 6 hrs in low-attachment plate (left, no shear stress) and after performing the clustering assay on the 6 hrs-settled aggregates for 1 hr (right, shear stress). **e,f**, Quantification of the number of single cells (**e**; *P*=0.7914, 0.0015) or number of clusters (**f**; *P*=0.9982, 0.0031) from WHIM12 samples shown in **d** (n=3 biological replicates). **g**, Transmission electron microscopy overview images of Ctrl and HAse treated LM2 cells settled for 16 hrs in a low-attachment plate. Images were taken at 500x magnification. See also Fig. 4b. **h,** Representative images (top) and quantification of the number of single cells (bottom) in MCF7 aggregates after shear stress exposure at various rotational speeds (n=4 biological replicates). Cells were settled for 24 hrs in a low-attachment plate (0 rpm) and sequentially agitated at each speed for 15 min. **i,** Western blots showing DSG2, DSC2 and DSP protein expression in LM2 control and corresponding knockdown cells. β-actin served as a loading control. **j,** Maximum intensity projections of fixed early LM2 clusters stained for HA (green) and either DSG2, DSC2 or DSP (red). Bottom panels depict grayscale images. White arrowheads indicate HA enrichment at the interaction sites. See also Fig. 4e for examples of fully mature clusters. Data are represented as mean ± SEM. Statistical significance: ns = not significant; ***P* = <0.01; *****P* = <0.0001 (ordinary one-way ANOVA (**b-c,e-f**)).

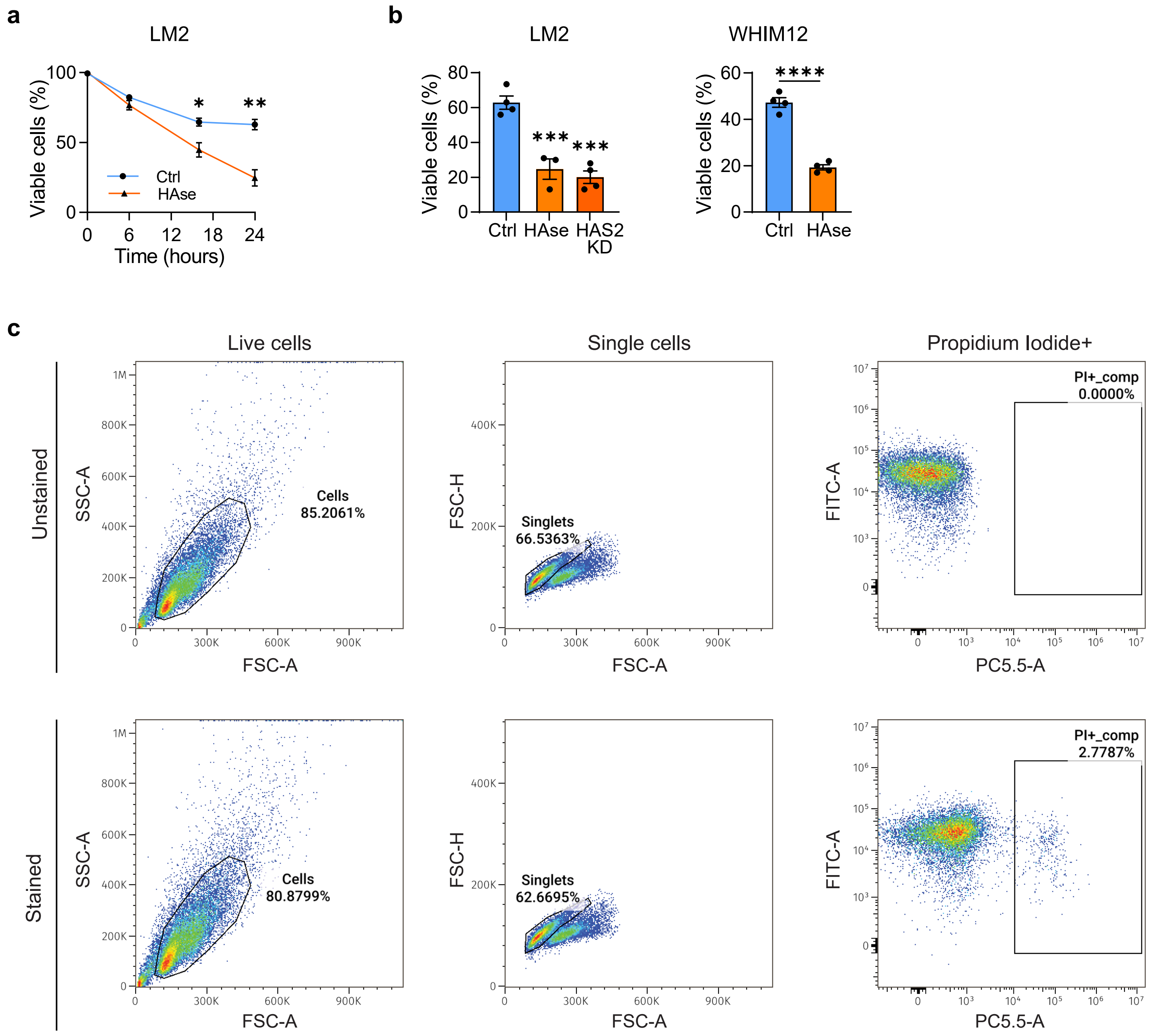
a
LM2
Viable cells (%)
100
50
0
Ctrl
HAse
*
**
0
6
12
18
24
Time (hours)
b
LM2
Viable cells (%)
80
60
40
20
0
***
***
Ctrl
HAse
HAS2 KD
WHIM12
Viable cells (%)
60
40
20
0
****
Ctrl
HAse
c
Live cells
Single cells
Propidium Iodide+
Unstained
Stained
SSC-A
FSC-A
Cells
85.2061%
80.8799%
FSC-H
Singlets
66.5363%
62.6695%
FITC-A
PC5.5-A
PI+_comp
0.0000%
2.7787%

Supplementary Figure 5. HA promotes cell survival under shear stress.

a, Time course showing percentage of alive LM2 Ctrl or HAse treated cells after 0, 6, 16, or 24 hrs of shear stress exposure (n=4 biological replicates; *P*=0.0158, 0.0022). **b**, Proportions of viable cells after 24 hrs of shear stress exposure. Left: LM2 Ctrl (n=4 biological replicates), HAse treated (n=3 biological replicates; *P*=0.0007), and HAS2 KD cells (n=4 biological replicates; *P*=0.0002) are shown. Right: WHIM12 PDX Ctrl or HAse treated cells (n=4 biological replicates) are shown. **c**, Gating strategy for **a-b** showing gates for live cells, single cells and PC5.5 (propidium iodide)-positive cells. Data are represented as mean ± SEM. Statistical significance: *P = <0.05; **P = <0.01; ***P = <0.001; ****P = <0.0001 (unpaired two-sided t test (**a**) or ordinary one-way ANOVA (**b**)).

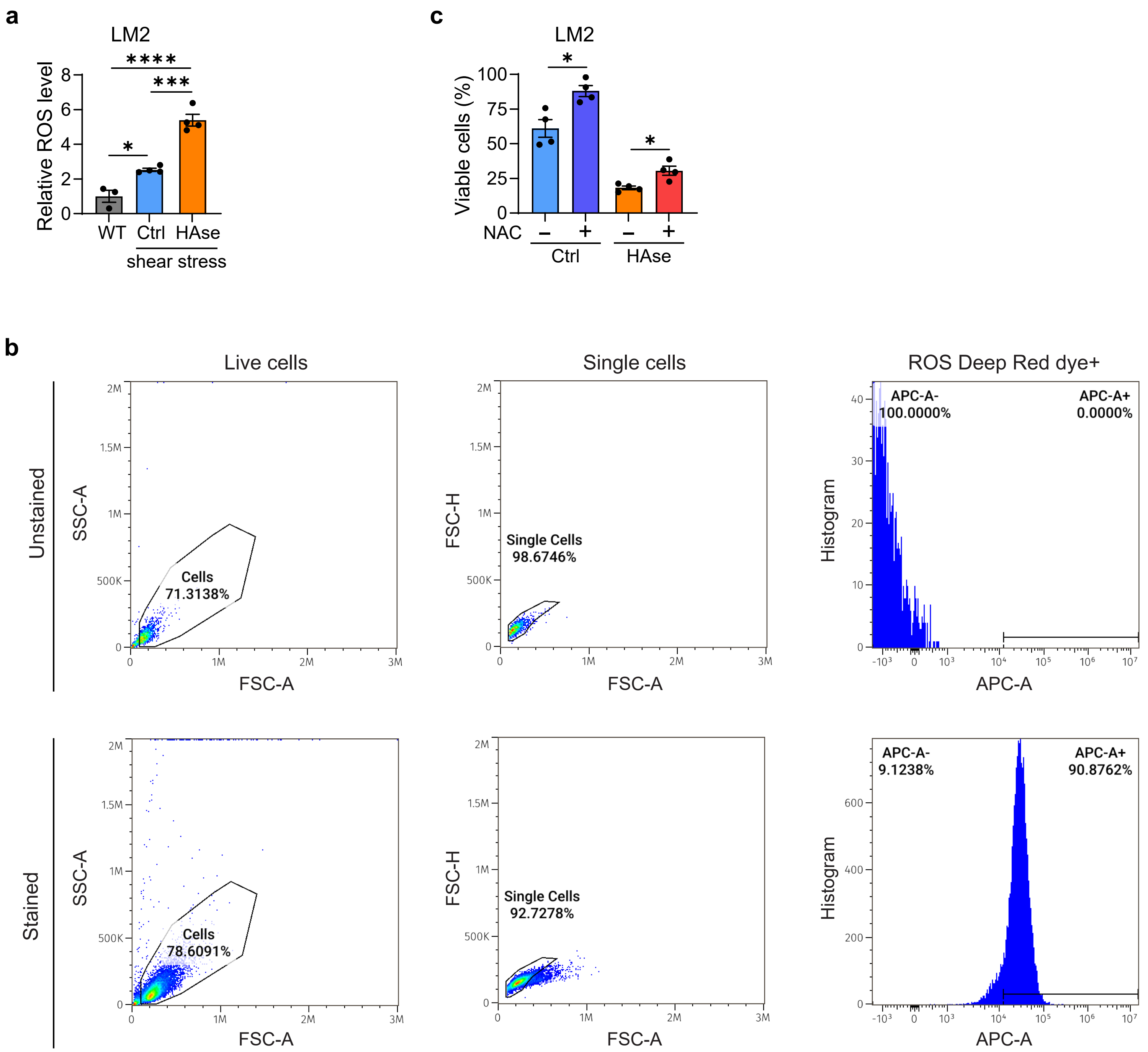
a
LM2
Relative ROS level
****
***
*
WT
Ctrl
HAse
shear stress
c
LM2
Viable cells (%)
*
*
NAC
Ctrl
HAse
b
Live cells
Single cells
ROS Deep Red dye+
Unstained
Stained
SSC-A
FSC-A
FSC-H
Histogram
APC-A
Cells
71.3138%
Single Cells
98.6746%
APC-A-
100.0000%
APC-A+
0.0000%
Cells
78.6091%
Single Cells
92.7278%
APC-A-
9.1238%
APC-A+
90.8762%

Supplementary Figure 6. HA protects from ROS upregulation and cell death under shear stress.

a, Relative ROS production in LM2 Ctrl or HAse treated cells after 12 hrs of shear stress exposure (n=3 (WT) or 4 (Ctrl, HAse) biological replicates; *P*=0.0134, 0.0002). Data are normalized to wild-type cells (WT) that were not exposed to shear stress. **b**, Gating strategy for **a** showing gates for live cells, single cells and APC (ROS Deep Red dye)-positive cells. **c**, Percentage of viable LM2 Ctrl or HAse-treated cells after 24 hrs of shear stress exposure, in the presence or absence of 5 mM of the antioxidant N-acetylcysteine (NAC) (n=4 biological replicates; *P*=0.0114, 0.0132). See Supplementary Fig. 5c for gating strategy. Data are represented as mean ± SEM. Statistical significance: *P = <0.05; ***P = <0.001; ****P = <0.0001 (ordinary one-way ANOVA (**a**) or unpaired two-sided t test (**c**)).

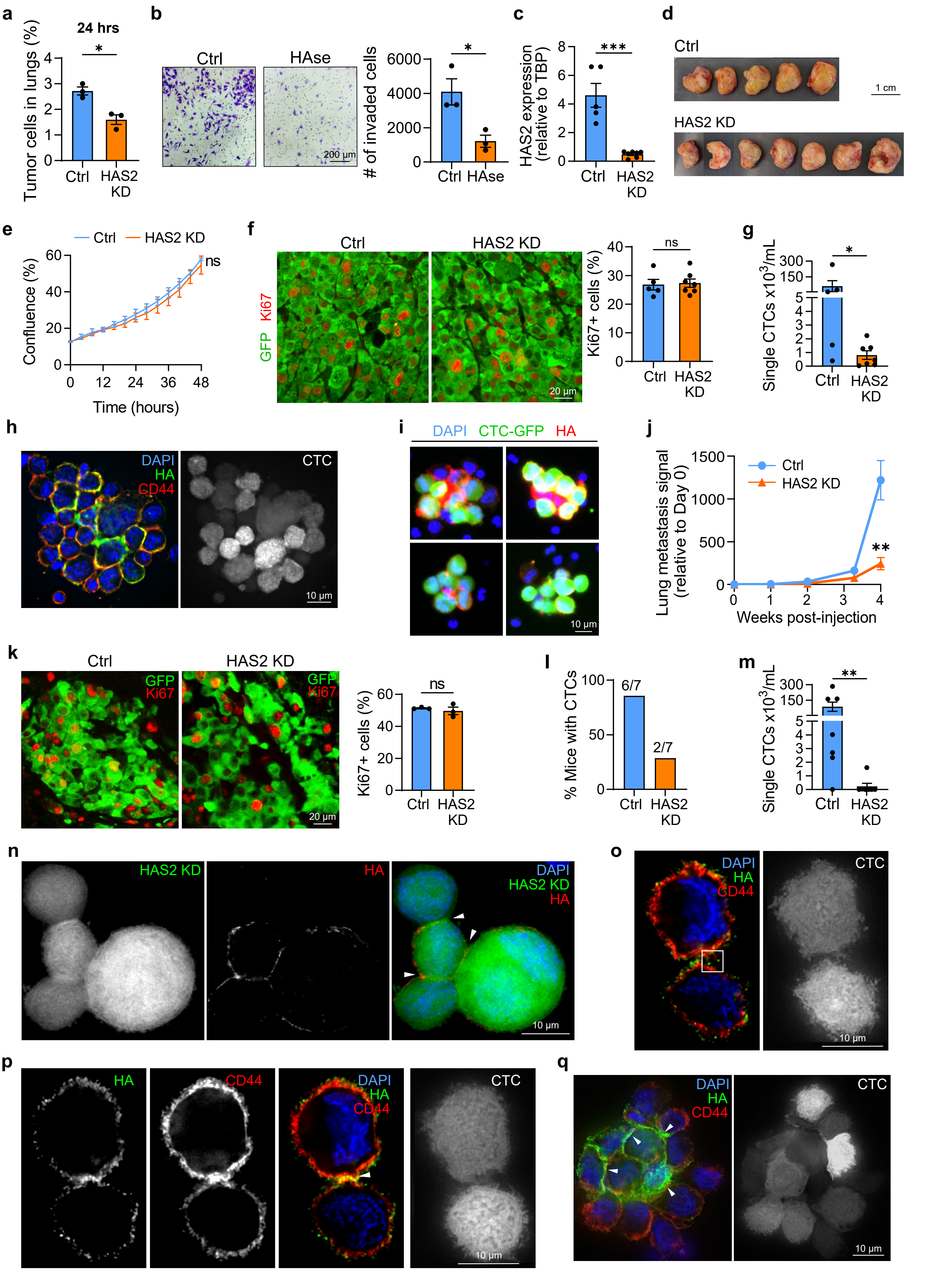
a
24 hrs
Tumor cells in lungs (%)
Ctrl
HAS2 KD
b
Ctrl
HAse
200 µm
# of invaded cells
Ctrl
HAse
c
HAS2 expression (relative to TBP)
Ctrl
HAS2 KD
d
Ctrl
HAS2 KD
1 cm
e
Ctrl
HAS2 KD
ns
Confluence (%)
Time (hours)
f
Ctrl
HAS2 KD
GFP Ki67
20 µm
Ki67+ cells (%)
g
Single CTCs x10^3/mL
h
DAPI
HA
CD44
CTC
10 µm
i
DAPI CTC-GFP HA
10 µm
j
Ctrl
HAS2 KD
Lung metastasis signal (relative to Day 0)
Weeks post-injection
k
Ctrl
HAS2 KD
GFP
Ki67
20 µm
Ki67+ cells (%)
l
% Mice with CTCs
6/7
2/7
Ctrl
HAS2 KD
m
Single CTCs x10^3/mL
n
HAS2 KD
HA
DAPI
10 µm
o
DAPI
HA
CD44
CTC
10 µm
p
HA
CD44
CTC
10 µm
q
DAPI
HA
CD44
CTC
10 µm

Supplementary Figure 7. HA is required for CTC clustering and metastasis *in vivo*.

a, Percentage of LM2 Ctrl and HAS2 KD tumor cells in the lungs 24 hrs after tail vein injection (n=3 mice per group; *P*=0.0106). **b**, Representative pictures (left) and quantification of LM2 Ctrl and HAse-treated cells that successfully invaded Matrigel chamber (right) after 24 hrs (n=3 biological replicates; *P*=0.0263). **c**, qRT-PCR results showing HAS2 mRNA expression in primary tumors from mice in Fig. 5a (n=5 Ctrl and 7 HAS2 KD mice; *P*=0.0002). Data is normalized to TBP. **d**, Images of primary tumors from mice in Fig. 5a, arranged by smallest to largest weight in each group. See also Fig. 5e. **e**, Quantification of the *in vitro* growth rate of Ctrl and HAS2 KD cells as determined by confluency (n=3 biological replicates; *P*=0.5634). **f**, Left: representative images of primary tumor sections from mice in Fig. 5a. GFP (green) and Ki67 (red) staining was used to detect proliferative tumor cells. Right: quantification of the percentage of Ki67-positive primary tumor cells (n=5 Ctrl and 7 HAS2 KD mice; *P*=0.8179). **g**, Quantification of single CTCs detected per mL of blood in mice in Fig. 5a (n=5 Ctrl and 7 HAS2 KD mice; *P*=0.048). See also Fig. 5g-h. **h**, Maximum intensity projection of fixed LM2 CTC cluster stained for HA (green pseudo color) and CD44 (red). CTCs (gray) were identified through the expression of the GFP tag. See also Fig. 5j. **i**, Additional examples of GFP-tagged CTC clusters (green) stained for HA (red). **j**, Quantification of lung metastasis signal across the four weeks of the experiment (n=7 mice per group; *P*=0.0016). Data is normalized to lung signal at time of injection. See also Fig. 5k-l. **k**, Left: representative images of lung sections from mice in Fig. 5k (n=3 mice per group). GFP (green) and Ki67 (red) staining was used to detect proliferative LM2 tumor cells. Right: quantification of the percentage of Ki67-positive tumor cells in lungs (*P*=0.5185). **l**, Percentage of mice from Fig. 5k in which CTCs were detected. **m**, Quantification of single CTCs detected per mL of blood in mice (n=7 mice per group; *P*=0.0064). See also Fig. 5n-o. **n**, Maximum intensity projection of a fixed mouse CTC cluster containing only GFP-tagged HAS2KD cells (green). HA channel (red) was imaged using the same settings as in Fig. 5r. The presence of two nuclei in the large cell suggests that it resulted from an abnormal mitosis. **o,p**, Single optical section of an early connection site in a LM2 CTC cluster stained for HA (green pseudo color) and CD44 (red). CTCs (gray) were identified through expression of a GFP tag. **p** depicts an alternative z-panel of Fig. 5q. White arrowheads indicate examples of HA enrichment. See Fig. 5q and Supplementary Movie 5. **q**, Maximum intensity projection of a large LM2 CTC cluster stained for HA (green pseudo color) and CD44 (red). CTCs (gray) were identified through the expression of the GFP tag. White arrowheads indicate examples of HA enrichment. See also Fig. 5r. Data are represented as mean ± SEM. Statistical significance: ns = not significant; *P = <0.05; **P = <0.01; ***P = <0.001 (unpaired two-sided t test (**a-c,e-f,j-k**) or two-sided Mann-Whitney test (**g,m**)). DAPI (blue) served as a nuclear counterstain. Note: for visualization purposes, HA staining was assigned a green pseudo-color to match the color scheme used throughout the manuscript.

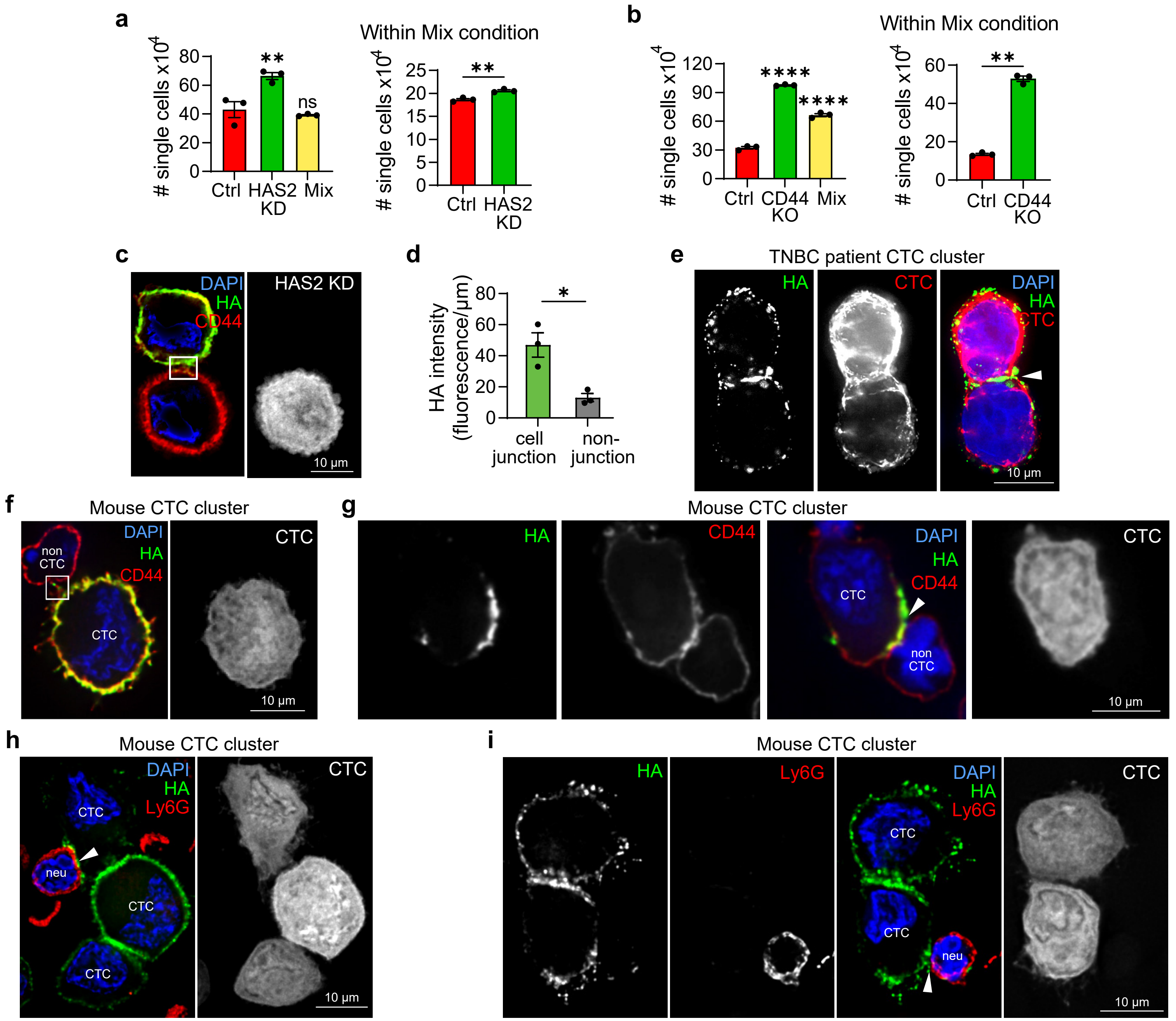
a
single cells x10^4
Ctrl
HAS2 KD
Mix
**
ns
Within Mix condition
b
CD44 KO
****
c
DAPI
HA
CD44
HAS2 KD
10 µm
d
HA intensity (fluorescence/µm)
cell junction
non-junction
*
e
TNBC patient CTC cluster
CTC
f
Mouse CTC cluster
non CTC
g
h
Ly6G
neu
i

Supplementary Figure 8. HA facilitates interactions between CTCs and non-CTCs.

a, Left: number of single cells remaining for each condition in Fig. 6a (n=3 biological replicates; *P*=0.0043, 0.5460). Right: Ctrl and HAS2 KD single cells remaining within the Mix condition in Fig. 6a (n=3 biological replicates; *P*=0.0012). **b**, Left: number of single cells remaining for each condition in Fig. 6b (n=3 biological replicates). Right: Ctrl and CD44 KO single cells remaining within the mix condition in Fig. 6b (n=3 biological replicates; *P*=0.0013). **c**, Maximum intensity projection of fixed early-interacting LM2 control (top) and HAS2 KD (bottom) cells stained for HA (green pseudo color) and CD44 (red). LM2 HAS2 KD cell was identified with a GFP tag (gray). See Fig. 6c. **d**, Quantification of HA signal at cell-cell interaction sites ("cell junction") and membrane areas that are not in contact with neighboring cells ("non-junction") for mixed LM2 cluster exemplified in Fig. 6d (n=3 biological replicates; *P*=0.0149). **e**, Maximum intensity projections of a TNBC patient CTC cluster stained for HA (green) and CTC markers (red). White arrowhead indicates HA enrichment at the CTC-CTC interaction site. **f,g**, Maximum intensity projections of fixed, heterotypic LM2 CTC clusters containing one CTC and one non-tumor cell. Cells were stained for HA (green) and CD44 (red pseudo color); RFP served as the CTC marker (gray). White arrowhead indicates the site of HA enrichment at the CTC and non-CTC interaction site in **g**. See also Fig. 6l. **h,i**, Maximum intensity projections of heterotypic CTC clusters containing two or three CTCs and one Ly6G-positive immune cell. Cells were labelled for HA (green) and Ly6G (red pseudo color); RFP served as the CTC marker (gray). "neu" denotes Ly6G+ neutrophils. White arrowheads indicate HA enrichment at the CTC-neutrophil interaction site. See also Fig. 6m. Data are represented as mean ± SEM. Statistical significance: ns = not significant; **P* = <0.05; ***P* = <0.01; *****P* = <0.0001 (paired two-sided t test (**a,b** *right*), unpaired two-sided t test (**d**) or ordinary one-way ANOVA (**a**,**b** *left*)). DAPI (blue) served as a nuclear counterstain. Note: for visualization purposes, green/red pseudo-color were assigned for HA/CD44/Ly6G to match the color scheme used throughout the manuscript.
