# Supplementary material for: Extracellular matrix mediates circulating tumor cell clustering in triple-negative breast cancer metastasis
Source: Nat Commun. 2026 Feb 6;17:1352. doi: 10.1038/s41467-026-69007-w (PMC12881594; doi:10.1038/s41467-026-69007-w)
Supplement: Supplementary file 2 — Description of Additional Supplementary Files [file 41467_2026_69007_MOESM2_ESM.pdf]

Description of Additional Supplementary Files

Supplementary Movie 1. Live imaging of early interaction events between two tumor cells, related to Fig. 3f.

Supplementary Movie 2. Live imaging of mid-late interaction events between two tumor cells, related to Supplementary Fig. 3e.

Supplementary Movie 3. Live imaging of cluster formation amongst multiple tumor cells, related to Fig. 3f and Supplementary Fig. 3e.

Supplementary Movie 4. Live imaging of interactions between control and CD44 KO cells, related to Fig. 3g.

Supplementary Movie 5. 3D walkthrough of an early CTC-CTC interaction site, related to Fig. 5q.
