# Supplementary material for: Extracellular matrix mediates circulating tumor cell clustering in triple-negative breast cancer metastasis
Source: Nat Commun. 2026 Feb 6;17:1352. doi: 10.1038/s41467-026-69007-w (PMC12881594; doi:10.1038/s41467-026-69007-w)
Supplement: Supplementary file 8 — Reporting Summary [file 41467_2026_69007_MOESM8_ESM.pdf]

nature portfolio

Corresponding author(s): Chonghui Cheng

Last updated by author(s): Dec 30, 2025

Reporting Summary

Nature Portfolio wishes to improve the reproducibility of the work that we publish. This form provides structure for consistency and transparency in reporting. For further information on Nature Portfolio policies, see our Editorial Policies and the Editorial Policy Checklist.

Statistics

For all statistical analyses, confirm that the following items are present in the figure legend, table legend, main text, or Methods section.

n/a	Confirmed	
☐	☒	The exact sample size (*n*) for each experimental group/condition, given as a discrete number and unit of measurement
☐	☒	A statement on whether measurements were taken from distinct samples or whether the same sample was measured repeatedly
☐	☒	The statistical test(s) used AND whether they are one- or two-sided<br>*Only common tests should be described solely by name; describe more complex techniques in the Methods section.*
☒	☐	A description of all covariates tested
☒	☐	A description of any assumptions or corrections, such as tests of normality and adjustment for multiple comparisons
☐	☒	A full description of the statistical parameters including central tendency (e.g. means) or other basic estimates (e.g. regression coefficient) AND variation (e.g. standard deviation) or associated estimates of uncertainty (e.g. confidence intervals)
☐	☒	For null hypothesis testing, the test statistic (e.g. *F*, *t*, *r*) with confidence intervals, effect sizes, degrees of freedom and *P* value noted<br>*Give P values as exact values whenever suitable.*
☒	☐	For Bayesian analysis, information on the choice of priors and Markov chain Monte Carlo settings
☒	☐	For hierarchical and complex designs, identification of the appropriate level for tests and full reporting of outcomes
☐	☒	Estimates of effect sizes (e.g. Cohen's *d*, Pearson's *r*), indicating how they were calculated

Our web collection on statistics for biologists contains articles on many of the points above.

Software and code

Policy information about availability of computer code

Data collection	No specific code was utilized for data collection.
Data analysis	Gene ontology (GO) cellular component analysis and GSEA KEGG pathway analysis were both conducted using WebGestalt (https://www.webgestalt.org) with the default setting. GSEA was performed with our in-house script. Fiji/ImageJ (v1.54.f) was used for image analysis.

For manuscripts utilizing custom algorithms or software that are central to the research but not yet described in published literature, software must be made available to editors and reviewers. We strongly encourage code deposition in a community repository (e.g. GitHub). See the Nature Portfolio guidelines for submitting code & software for further information.

Data

Policy information about availability of data

All manuscripts must include a data availability statement. This statement should provide the following information, where applicable:

- Accession codes, unique identifiers, or web links for publicly available datasets
- A description of any restrictions on data availability
- For clinical datasets or third party data, please ensure that the statement adheres to our policy

Source Data are provided with this paper and all the data that support the findings of this study are available within the Article and its Supplementary Information. Further information and requests for resources and reagents should be directed to the lead contact, Chonghui Cheng (Chonghui.Cheng@BCM.edu). No large-scale datasets or custom code were generated or analyzed in this study. Publicly available datasets:TCGA BRCA RNAseq expression dataset was downloaded from the GDC portal (https://portal.gdc.cancer.gov). METABRIC breast cancer expression dataset was downloaded from the cBioPortal (https://www.cbioportal.org). CCLE

expression dataset was downloaded from Broad Institute's DepMap Portal (https://depmap.org/portal).

Research involving human participants, their data, or biological material

Policy information about studies with human participants or human data. See also policy information about sex, gender (identity/presentation), and sexual orientation and race, ethnicity and racism.

Reporting on sex and gender	Breast cancer primarily occurs in females, so human samples from female breast cancer patients were utilized in this study.
Reporting on race, ethnicity, or other socially relevant groupings	No such groupings were used.
Population characteristics	Blood samples from female patients with metastatic triple-negative breast cancer were analyzed for the presence of CTC clusters.
Recruitment	De-identified samples were obtained from the Lester and Sue Smith Breast Cancer Center at Baylor College of Medicine.
Ethics oversight	Protocol was approved by IRB committee at Baylor College of Medicine.

Note that full information on the approval of the study protocol must also be provided in the manuscript.

Field-specific reporting

Please select the one below that is the best fit for your research. If you are not sure, read the appropriate sections before making your selection.

[x] Life sciences [ ] Behavioural & social sciences [ ] Ecological, evolutionary & environmental sciences

For a reference copy of the document with all sections, see nature.com/documents/nr-reporting-summary-flat.pdf

Life sciences study design

All studies must disclose on these points even when the disclosure is negative.

Sample size	Mouse experiments were performed with groups of 7-8 mice based on pilot experiments and to account for any mortality prior to the completion of the experiment.
Data exclusions	No data was excluded except in the event of animal mortality prior to completion of the experiment.
Replication	All experiments were replicated at least three times and successfully reproduced.
Randomization	Mice and cell line samples were randomly allocated into groups to minimize bias.
Blinding	Blinding was performed for all mouse experiments, both during data collection and analysis. Blinding was not performed for molecular assays including qPCR, western blotting, cluster assay, flow cytometry, and microscopy of cell culture cells as these experiments utilized objective, automated readouts and/or predefined analysis criteria that were consistently applied across all samples.

Reporting for specific materials, systems and methods

We require information from authors about some types of materials, experimental systems and methods used in many studies. Here, indicate whether each material, system or method listed is relevant to your study. If you are not sure if a list item applies to your research, read the appropriate section before selecting a response.

Materials & experimental systems

n/a	Involved in the study	
[ ]	[x]	Antibodies
[ ]	[x]	Eukaryotic cell lines
[x]	[ ]	Palaeontology and archaeology
[ ]	[x]	Animals and other organisms
[x]	[ ]	Clinical data
[x]	[ ]	Dual use research of concern
[x]	[ ]	Plants

Methods

n/a	Involved in the study	
[x]	[ ]	ChIP-seq
[ ]	[x]	Flow cytometry
[x]	[ ]	MRI-based neuroimaging

Antibodies

Antibodies used	For immunofluorescence, the following antibodies were used: CD44-IM7 (Santa Cruz Biotechnology sc-18849; 1:100), DSG2 (Proteintech 21880; 1:100), DSC2 (ABclonal A10211; 1:100), DSP (ABclonal A7635; 1:100), Tubulin (Proteintech 11224-1-AP; 1:100),

	Ki67 (eBioscience 14-5698-82; 1:100), GFP (Abcam ab13970; 1:100), RFP (Rockland 600-401-379; 1:100), Ly6G-AF594 (Biolegend 127636; 1:50), Pan-cytokeratin (Santa Cruz Biotechnology sc-8018; 1:100), Cytokeratin 19 (Invitrogen MA5-12663; 1:100), EpCAM-AF488 (Cell Signaling 5198S; 1:50), and EGFR-AF488 (Biolegend 352908; 1:50). Biotinylated HA binding protein (Millipore 385911; 2 µg/mL) was used to detect HA and AF568-coupled phalloidin (Thermo Fisher A12380; 1:100) to detect actin.<br><br>For immunoblotting, the following antibodies were used: CD44 (R&D BBA10; 1:1000), HA tag (Cell Signaling 3724S; 1:1000), E-cadherin (Cell Signaling 3195S; 1:2000), HAS2 (Santa Cruz Biotechnology sc-514737; 1:500), DSG2 (Proteintech 21880; 1:1000), DSC2 (ABclonal A10211; 1:1000), DSP (ABclonal A7635; 1:1000), GAPDH (Millipore MAB374; 1:3000), and β-actin (Sigma A1978; 1:3000).
Validation	These antibodies have been validated by the manufacturers and/or published literature. We also utilized knockdown/overexpression samples to confirm the specificity in Supplementary Fig. 2c, 2h, 2m, 2r, and 4i.

Eukaryotic cell lines

Policy information about cell lines and Sex and Gender in Research

Cell line source(s)	The following normal or cancer cell lines were used, with sources indicated in parenthesis. 293FT (ATCC), BT549 (ATCC), MDA-MB-231 and its derivative LM2 (Yibin Kang), HMLE (Jing Yang), MCF10A (Alexander Minella), BT474 (Charles V. Clevenger), SUM159 (Robert Weinberg), WHIM12 (Matthew Ellis), MCF7 (Marcus Peter), SKBR3 (Vince Cryns), DAOY (Richard Hurwitz), PC-3 (Raymond Bergan), and PANC-1 (BCM Molecular and Cellular Biology Tissue Culture Core Laboratory). DAOY, PC-3 and PANC-1 are established from male donors, while the remaining cell lines are from female donors.
Authentication	Cell lines used have not been authenticated since being received by the lab. Low-passage cells were used for all experiments.
Mycoplasma contamination	All cell lines tested negative for mycoplasma.
Commonly misidentified lines (See ICLAC register)	None of the commonly misidentified cell lines were used.

Animals and other research organisms

Policy information about studies involving animals; ARRIVE guidelines recommended for reporting animal research, and Sex and Gender in Research

Laboratory animals	Species: Mus musculus; Strain: NSG (NOD.Cg-Prkdcscid Il2rgtm1Wjl/SzJ); Age: 6-9 weeks. Mice were obtained from Jackson Laboratory and subsequently bred in-house. All mice were housed in a 14:10 hrs light/dark cycle at an ambient temperature of 20-23°C. Humidity was maintained between 30-70%.
Wild animals	No wild animals were used.
Reporting on sex	Breast cancer is a disease that primarily affects females so animals used were female and sex was not considered beyond this determination. All results are likely applicable to male subjects as nothing was excluded from consideration on the basis of sex.
Field-collected samples	No field-collected samples were used.
Ethics oversight	All animal procedures were performed with approval from the Institutional Animal Care and Use Committee at Baylor College of Medicine (Protocol number AN-7145).

Note that full information on the approval of the study protocol must also be provided in the manuscript.

Plants

Seed stocks	*Report on the source of all seed stocks or other plant material used. If applicable, state the seed stock centre and catalogue number. If plant specimens were collected from the field, describe the collection location, date and sampling procedures.*
Novel plant genotypes	*Describe the methods by which all novel plant genotypes were produced. This includes those generated by transgenic approaches, gene editing, chemical/radiation-based mutagenesis and hybridization. For transgenic lines, describe the transformation method, the number of independent lines analyzed and the generation upon which experiments were performed. For gene-edited lines, describe the editor used, the endogenous sequence targeted for editing, the targeting guide RNA sequence (if applicable) and how the editor was applied.*
Authentication	*Describe any authentication procedures for each seed stock used or novel genotype generated. Describe any experiments used to assess the effect of a mutation and, where applicable, how potential secondary effects (e.g. second site T-DNA insertions, mosiacism, off-target gene editing) were examined.*

Flow Cytometry

Plots

Confirm that:

- [x] The axis labels state the marker and fluorochrome used (e.g. CD4-FITC).
- [x] The axis scales are clearly visible. Include numbers along axes only for bottom left plot of group (a 'group' is an analysis of identical markers).
- [x] All plots are contour plots with outliers or pseudocolor plots.
- [x] A numerical value for number of cells or percentage (with statistics) is provided.

Methodology

Sample preparation	Cells were detached using TrypLE Express and resuspended in DMEM supplemented with 0.5% FBS. 100K cells in 500 µl of DMEM supplemented with 0.5% FBS and either DMSO or HAse were seeded in each well of a 24 well plate, which was immediately placed on an orbital shaker at 37°C in 5% CO2. Cells were agitated at 200 rpm for 6, 16, or 24 hrs. For each condition, cells from 3 wells were combined (to obtain enough material) at each time point and spun down in flow tubes at 500g for 5 min. Pellets were resuspended into a single cell suspension in PBS and stained with Propidium Iodide (PI; BD Pharmingen 556463) for 15 min at RT in the dark. For ROS measurement, cells were prepared similarly, but they were agitated for 12 hrs prior to collection. Cells were incubated in PBS containing ROS Deep Red dye (Abcam ab186029; 1:1000) for 30 min at 37ºC in the dark.
Instrument	Samples were run on CytoFLEX benchtop flow cytometer (Beckman Coulter) to assess PI staining or determine ROS levels.
Software	Collection: Beckman Coulter CytExpert v2.4.0.28; Analysis: FlowJo software v10
Cell population abundance	Cell fractions were not sorted in this study.
Gating strategy	FSC-A vs. SSC-A was gated to exclude debris, followed by FSC-A vs. FSC-H to exclude cell aggregates. For PI staining, the PC5.5-negative population was calculated to be "viable". For ROS staining, the APC+ population was calculated to be ROS+. In both experiments, positive and negative fluorescence gates were determined using matched negative (unstained) and positive control samples.

- [x] Tick this box to confirm that a figure exemplifying the gating strategy is provided in the Supplementary Information.
